# Triterpenes and Aromatic Meroterpenoids with Antioxidant Activity and Neuroprotective Effects from *Ganoderma lucidum*

**DOI:** 10.3390/molecules24234353

**Published:** 2019-11-28

**Authors:** Cuifang Wang, Xuemin Liu, Chenlei Lian, Jiaying Ke, Jieqing Liu

**Affiliations:** 1 College of Oceanology and Food Science, Quanzhou Normal University, Quanzhou 362000, China; kejiaying2003@e-mail.com; 2 School of Medicine, Huaqiao University, Quanzhou 362021, Chinaliancl@hqu.edu.cn (C.L.)

**Keywords:** antioxidant activity, *Ganoderma lucidum*, methyl ganoderate G1, neuroprotective effects

## Abstract

Reactive oxygen/nitrogen species generated in the human body can cause oxidative damage associated with many degenerative diseases such as atherosclerosis, dementia, coronary heart diseases, aging, and cancer. There is a great interest in developing new antioxidants from *Ganoderma* fungus due to its low toxicity. As part of our ongoing search for antioxidative constituents from the fruiting bodies of *Ganoderma lucidum*, the chemical constituents were investigated and seven secondary metabolites, including one new lanostane triterpene (**1**), two known aromatic meroterpenoids (**6**–**7**), and four known triterpenes (**2**–**5**), were isolated by a series of chromatographic methods. The structures of the seven compounds were elucidated by spectroscopic techniques. The isolated compounds were tested in vitro for antioxidant potencies and neuroprotective activities against H_2_O_2_ and aged Aβ-induced cell death in SH-SY5Y cells. As a result, compounds **1**, **6**, and **7** exhibited potent antioxidant and neuroprotective activities. Additionally, all isolated compounds were tested for radical scavenging activities. Compounds **6** and **7** showed the comparable free radical scavenging activities with the standard drug in both ABTS (2, 2’-azobis (3-ethylbenzothiazole-6-sulfonaic acid)) and ORAC (oxygen radical absorbance capacity) experiments. The results from this study suggested that *G. lucidum* and its metabolites (especially the meroterpenoids) may be potential functional food ingredients for the antioxidation and prevention of neurogenerative diseases.

## 1. Introduction

Reactive oxygen species (ROS) can cause extensive damage to DNA, proteins, and lipids. This could be the fundamental cause of aging and many other important diseases such as cancer, cardiovascular diseases, and neurogeneration [1]. Deposition of different physicochemical forms of amyloid β peptide (Aβ) constitutes a major neuropathological hallmark of Alzheimer’s disease [2]. Several lines of evidence suggest that Aβ may exert its pathological effects in the central nervous system at least in part through ROS-mediated mechanisms [3,4]. Owing to the increased demand for and importance of antioxidants in day-to-day life, the search for effective, nontoxic, natural compounds with antioxidant activity has increasingly become a matter of interest.

A large number of medicinal mushrooms have recently been reported to possess significant antioxidant activity [5,6]. *Ganoderma lucidum* is known as the “mushroom of immortality” and considered to be a panacea to cure all kinds of diseases in Chinese folklore. As demonstrated by the numerous publications, polysaccharides are important contributors to the antioxidant properties reported for *Ganoderma* species [7,8,9]. Recently, research found that the total phenols of *Ganoderma lucidum* also showed significant antioxidant activity [10]. Several meroterpenoids from *Ganoderma cochlear* and *Ganoderma capense* were isolated for their antioxidant activities [11,12]. The aromatic meroterpenoids have attracted the attention of many phytochemists, chemists, and pharmocologists, due to their diverse structures and significant bioactivities (antioxidant, anti-HIV protease, and antifibrotic activities) [13,14,15,16]. Meanwhile, some oxygenated lanostane triterpenoids also showed potent free radical scavenging activities and neuroprotective activity [17,18]. These previous studies suggested *Ganoderm* mushrooms might be a novel resource for natural antioxidant exploration.

Our group has long been interested in *Ganoderma* species [19,20,21] and our previous studies showed that the total ethanol extract of *G. lucidum* displayed antioxidant activity. As part of our ongoing search for antioxidative constituents from this fungus, seven compounds, including a new lanostanoid, four known lanostainoids, and two known aromatic meroterpenoids, were isolated from the fruiting bodies of *G. lucidum*. Their antioxidant potencies were investigated in various systems. Meanwhile, compounds **1**, **6**, and **7** were evaluated for their neuroprotective properties. Our results will lay the foundation for the further in vivo bioactive research and provide a theoretical basis to the application of *G. lucidum* on antioxidation and antineurological disease.

## 2. Results

### 2.1. Structure Elucidation of Compound 1

**Figure 1:**
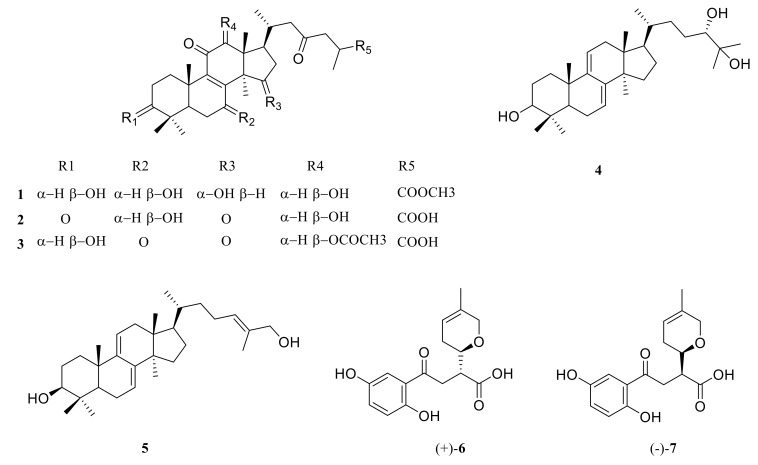
Structures of isolate compounds from *Ganoderma lucidum*.

The 95% ethanol extracts of the fruiting bodies of *G. lucidum* were fractionated and purified by a series of chromatographic methods to obtain one new lanostanoid and six known compounds (**1**–**7**, Figure 1).

Compound **1** was obtained as white powder (CH_2_Cl_2_) with a molecular formula of C_31_H_48_O_8_ from the molecular ion peak [M+Na]^+^ at *m*/*z* 571.3241 (calcd for C_31_H_48_O_8_Na 571.3247) in the high resolution electrospray ionization mass spectroscopy (HRESIMS). The ^1^H nuclear magnetic resonance (NMR) spectrum (Table 1) of **1** showed the presence of five tertiary methyl signals at *δ* 0.86 (s), 0.95 (s), 1.04 (s), 1.26 (s), 1.27 (s), and two doublet methyl signals at *δ* 0.89 (d, *J* = 6.4 Hz) and 1.19 (d, *J* = 7.1 Hz). The ^13^C NMR and distortionless enhancement by polarization transfer (DEPT) spectra (Table 1) of **1** revealed 31 carbon signals, including eight methyls, six methylenes, eight methines, and nine quaternary carbons (three carbonyls). These NMR characteristics were similar to those of methyl ganoderate G [22]. Comparison of the ^13^C NMR data of **1** with those of methyl ganoderate G showed that the hydroxyl group (*δ* 72.48) in **1** replaced the carbonyl group (*δ* 216.8) in methyl ganoderate G at C-15, which was confirmed by the heteronuclear multiple bond correlation (HMBC) correlations of H-15 with C-16 and C-14; of Me-30 with C-15 (Figure S7 in Supplementary Material). In the rotating frame overhauser effect spectroscopy (ROESY) spectrum, cross-peak of H-3/H-5, H-5/H-7, Me-29/H-3, Me-30/H-12, and Me-18/H-15 indicated that 3-OH, 7-OH, 12-OH, and 15-OH were β-oriented, β-oriented, β-oriented, and α-oriented, respectively (Figure S6 in Supplementary Material). Thus, the structure of compound **1** was 3β, 7β, 12β, 15α-tetrahydroxy-11, 23-dioxo-5α-lanost-8-en-26-oate, named methyl ganoderate G1 (**1**). 

The known isolates were identified by comparing their physical and spectroscopic data with literature data. They were ganoderic acid D2 (**2**) [23], ganoderic acid H (**3**) [23], ludidumol B (**4**) [24], ganoderiol B (**5**) [25], lingzhine E (**6**) [26], and lingzhine F (**7**) [26], by comparison of their NMR data with those in the literature. 

### 2.2. Bioactivity Evaluation

#### 2.2.1. ABTS^.+^ Radical Scavenging Assay

The ABTS^.+^ assay is an excellent tool for determining the antioxidant activity of hydrogen-donating antioxidants and of chain-breaking antioxidants [27]. In the present study, the ability of test samples to scavenge ABTS was assessed on the basis of their EC_50_ values, defined above as an effective concentration at which the ABTS radical was scavenged by 50%. EC_50_ values of the isolates and trolox (used as a reference compound) are given in Table 2. A low EC_50_ value indicates strong antioxidant activity in a tested sample. Among these compounds, lingzhine E (**6**) and lingzhine F (**7**) showed comparable ABTS^.+^ scavenging effects with EC_50_ values of 0.59 ± 0.15 and 0.27 ± 0.05 mM, respectively, which was close to the positive control (trolox) with an EC_50_ value of 0.42 ± 0.03 mM.

#### 2.2.2. ORAC Antioxidant Activity Assay

ORAC is also a widely used in vitro antioxidant capacity assay [28]. It is a chemical antioxidant assay that is based on the inhibition of the peroxyl-radical induced oxidation initiated by the thermal decomposition of 2, 2’-azobis-(2-amidinopropane) dihydrochloride (AAPH). The antioxidant capacity of the isolates was also measured by ORAC assay and the potency of the natural compound was compared with that of the positive control, quercetin, which is well known for its use as an antioxidant. The ORAC results are expressed as trolox equivalent [4] and shown in Table 2. A high ORAC value indicates strong antioxidant activity in a tested sample. Among the seven compounds, the more potent radical scavenger was lingzhine E (**7**) (7.24 ± 0.27 μmol TE/μmol) with a similar value to quercetin (7.78 ± 0.27 μmol TE/μmol), followed by lingzhine (**6**) (5.42 ± 0.20 μmol TE/μmol). This result was consistent with those of the ABTS assay.

#### 2.2.3. Antioxidant Effects on H_2_O_2_-Induced ROS Production in SH-SY5Y Cells

**Figure 2:**
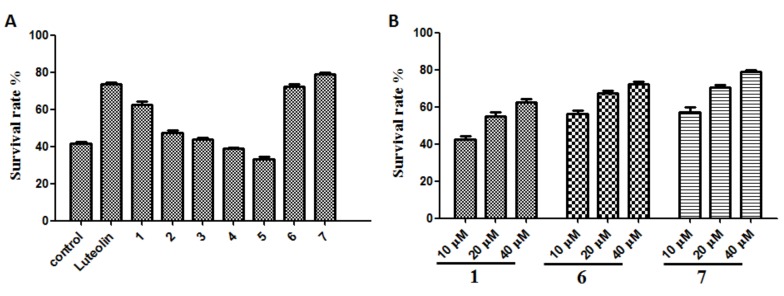
Effect of compounds **1**–**7** on H_2_O_2_-induced SH-SY5Y cell death. (**A**) **1**–**7**: compounds **1**–**7** 40 μM; control: 200 μM H_2_O_2_ as negative control; Luteolin: 40 μM luteolin as positive control; (**B**) the survival rate of compounds **1**, **6**, and **7** in different concentrations. Data represent the mean values of four experiments ± SD.

**Figure 3:**
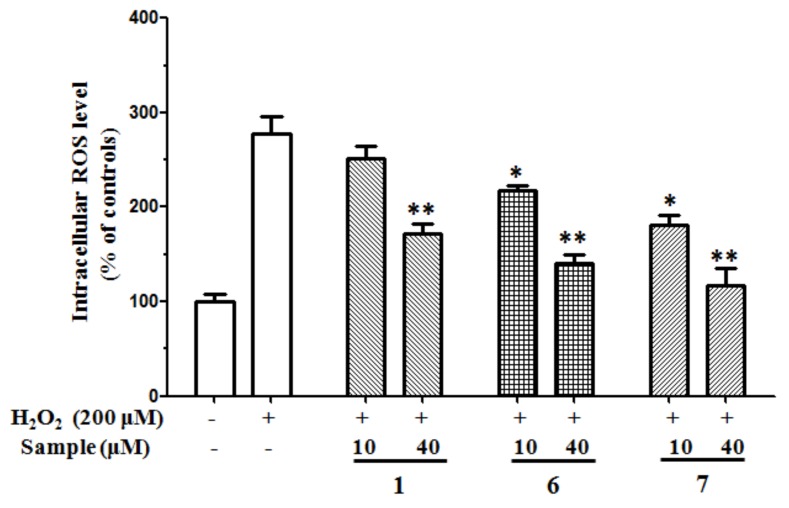
Effects of compounds **1**, **6**, and **7** on DCFH oxidation induced by H_2_O_2_ in SH-SY5Y cells. The columns represent % of change in fluorescence intensity with respect to H_2_O_2_-treated cells. All data were expressed as mean ± S.D., *n* = 4. **p < 0.01, *p < 0.05 compared with H_2_O_2_-treated control.

The in vitro antioxidant assays, based on chemical reactions, are easy to operate and widely used to evaluate antioxidant capacities. However, their main disadvantage is that they do not reflect cellular physiological conditions. Therefore, a cell-based antioxidant activity assay to evaluate the antioxidant potential is necessary. ROS, such as the superoxide anion radical, hydrogen peroxide, and hydroxyl radical, are generated during many physiological and pathological processes and reported to function in an array of intracellular signaling cascades [29]. H_2_O_2_ is used extensively as an inducer of oxidative stress in vitro because its cellular actions and pathophysiological roles have been well studied [30]. SH-SY5Y human neuroblastoma cells are highly sensitive to oxidative stressors such as H_2_O_2_. The protective activity of compounds **1**–**7** against H_2_O_2_-induced oxidative stress was evaluated on SH-SY5Y cells at the concentration of 40 μM. After incubation with 200 μM H_2_O_2_ for 12 h, only 41.54 ± 2.04% of cultured cells survived. Compounds **1**, **6**, and **7** (10–40 μM) could protect H_2_O_2_-induced cell damage in a dose-dependent relationship, and the survival rates at 40 μM were 62.68 ± 2.81%, 72.57 ± 2.12%, and 78.96 ± 1.86%, respectively. Luteolin is used as positive control with a survival rate of 73.59 ± 2.19% at 40 μM (Figure 2). 2’,-7’-dichlorofluorescin diacetate (DCFH-DA) is one of the most widely used techniques for directly measuring the redox state of a cell. When SH-SY5Y cells were challenged with 200 μM H_2_O_2_, ROS were generated over twofold compared to the unchallenged control, while the treatment with compounds **1**, **6**, and **7** dose-dependently decreased the H_2_O_2_-mediated ROS formation (Figure 3). These results imply that methyl ganoderate G1 (**1**), lingzhine E (**6**), and lingzhine F (**7**) may have an ability to directly scavenger ROS and/or free radicals.

#### 2.2.4. Protection of SH-SY5Y Cells Against Aβ-Induced Damage

**Figure 4:**
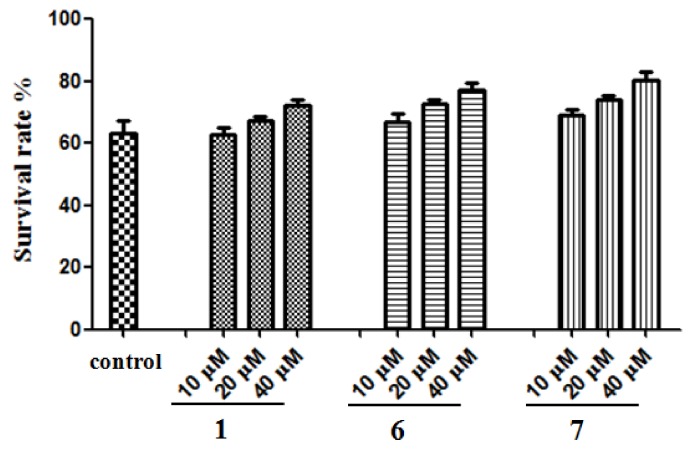
Effect of compounds **1**, **6**, and **7** on aged Aβ_25–35_-induced SH-SY5Y neurotoxicity. Control: 25 μM aged Aβ_25–35_ as negative control. Data represent the mean values of four experiments ± SD.

There is abundant evidence suggesting that an excess of Aβ, which aggregates into toxic fibrillar deposits, plays a central role in the etiology of Alzheimer’s disease (AD) [31]. In support of this hypothesis, numerous in vitro and in vivo studies have reported on the neurotoxic effects of Aβ-related fragments in neurons derived from regions severely affected in AD [32]. Although the precise mechanisms mediating the toxic properties of Aβ have yet to be extensively understood, it has been proposed that they are associated with oxidative stress-dependent apoptosis [33]. Hence, the capacity of methyl ganoderate G1 (**1**), lingzhine E (**6**), and lingzhine F (**7**) in protecting neuroblastoma SH-SY5Y cells against Aβ-induced damage was examined. Aβ_25__–__35_, a synthetic peptide that possesses most of the physical and biological properties of full-length Aβ and is often used to study the neuroprotective effects of various compounds, was predicted to modulate Aβ toxicity in vitro [34]. In this study, the neuroprotective activity of compounds **1**, **6**, and **7** against Aβ-induced oxidative stress was evaluated on SH-SY5Y cells. After incubation with 25 μM aged Aβ_25__–__35_ for 24 h, only 63.43 ± 4.81% of cultured cells survived. Treatment of compounds **1**, **6**, and **7** (10–40 μM) could protect Aβ-induced cell damage in a dose-dependent relationship, and the survival rates at 40 μM were 72.4 ± 3.19%, 77.11 ± 4.18%, and 80.17 ± 5.19%, respectively (Figure 4).

#### 2.2.5. Inhibition of ROS Generation Induced by Aβ_25__–__35_ in SH-SY5Y Cells

**Figure 5:**
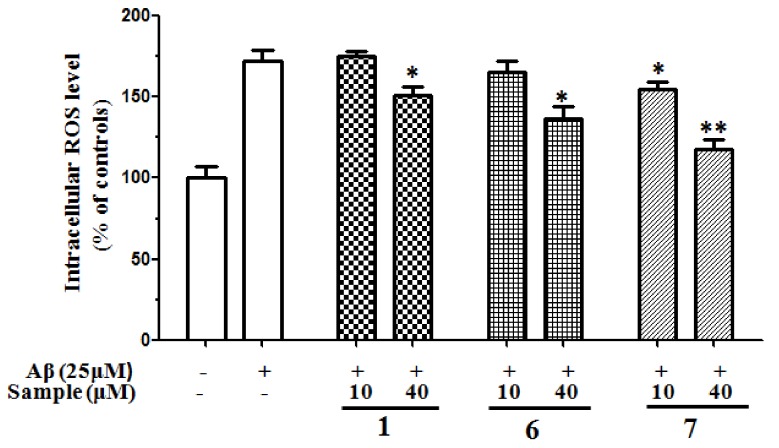
Effects of compounds **1**, **6**, and **7** on DCFH oxidation induced by Aβ_25__–35_ in SH-SY5Y cells. The columns represent % of change in fluorescence intensity with respect to Aβ_25__–35_-treated cells. All data were expressed as mean ± S.D., *n* = 4. **p < 0.01, *p < 0.05 compared with Aβ_25__–35_-treated control.

The neurotoxicity of Aβ has been reported to be mediated with oxygen free radicals and attenuated by antioxidants and free radical scavengers. Many reports have demonstrated the involvement of ROS formation in Aβ-induced neurotoxicity [35]. We investigated whether compounds **1**, **6**, and **7** affect ROS formation by Aβ using DCFH-DA probe staining. As shown in Figure 5, exposure to Aβ induced an elevation of the intracellular ROS levels. Treatment with compounds **1**, **6**, and **7** ameliorated the intracellular ROS elevation. These results indicated that methyl ganoderate G1 (**1**), lingzhine E (**6**), and lingzhine F (**7**) have the ability to scavenge Aβ-induced ROS increase.

## 3. Discussion

Since 2000, many structurally diverse aromatic meroterpenoids were found from different species of *Ganoderma* and have attracted the interest of chemists and pharmacologists [11,15,26]. In the present study, two aromatic meroterpenoids (**6**–**7**) were isolated and identified from the fruiting bodies of *G. lucidum*. Our previous and present research exhibited that aromatic meroterpenoids had excellent in vitro antioxidant effects [11]. Hence, the antioxidant potencies of the two compounds were first investigated employing various in vitro systems. The results showed that lingzhine E (**6**) and lingzhine F (**7**) had strong antioxidant activities. Moreover, the lanostanoid triterpenes obtained from the *Ganoderma* family have been reported to show strong antioxidant activities and neuroprotective activities [17,18]. Hence, the isolated compounds were evaluated in vitro for their antioxidant potencies and neuroprotective activities against H_2_O_2_- and Aβ-induced cell death in SH-SY5Y cells. Methyl ganoderate (**1**), lingzhine E (**6**), and lingzhine F (**7**) possessed significant neuroprotective activities. These results will lay the foundation for further in vivo bioactive research and provide a theoretical basis to the application of *G. lucidum* on antineurological disease.

## 4. Materials and Methods

### 4.1. Materials and Reagent

The fruiting bodies of *G. lucidum* were purchased from Wuyi Mountain Traditional Chinese Medicine Market in Fujian Province of China in June 2016. The mushroom was identified by Prof. Chen Tiqiang, who works at Fujian Academy of Agricultural Sciences.

ABTS detection kit and Cell Counting Kit (CCK-8) were purchased from Beyotime Institute of Biotechnology (Nanjing, China). 2, 2’-Azobis-(2-amidinopropane) dihydrochloride (AAPH), 6-hydroxy-2,5,7,8-tetramethylchroman-2-carboxylic acid (trolox), fluorescein sodium salt (FL), 2’,7’-dichlorofluorescein diacetate (DCFH_2_-DA), dimethyl sulfoxide (DMSO), and amyloid β-Protein Fragment 35-25 (Aβ_25__–35_) were purchased from Sigma-Aldrich, Inc. (St. Louis, MO, USA). Minimum essential media (MEM) and F12 were purchased from Gibco BRL (Life Technologies, China). Trypsin-EDTA and penicillin–streptomycin solution were acquired from Gibco BRL (Grand Island, NY, USA). Fetal bovine serum (FBS) was obtained from Corning (Mediatech, Manassas, VA, USA). The organic solvents used for fraction and separation were purchased from Sinopharm Chemical Reagent Co. Ltd. (Shanghai, China).

### 4.2. Apparatus and Chemicals

1D and 2D NMR spectra were recorded in CDCl_3_ using a Bruker AVANCE III-600 spectrometer (Bruker Corp., Switzerland), and tetramethyl silane (TMS) was used as an internal standard. Chemical shifts (δ) were expressed in ppm with reference to TMS. Optical rotations were obtained with a Jasco P-1020 polarimeter (Japan). HRESIMS spectral data were recorded on an ultra performance liquid chromatography-time of flight-mass spectrometry (UPLC-TOF-MS) Waters, MA, USA), carried out on Waters Acquity UPLC columns at 35 °C (Zorbax eclipse plus C18: 2.1 mm × 50 mm, 1.8 μm). Column chromatography was conducted using silica gel (Qingdao Marine Chemistry Company, China) and Sephadex LH-20 (Pharmacia, Sweden). Activity data were recorded on a microplate reader (Infinite M200 PRO, Tecan, Sweden).

### 4.3. Extraction and Isolation

The dried fruiting bodies of *G. lucidum* (30 kg) were powdered and extracted with 95% ethanol three times at room temperature. After filtration and evaporation in vacuo, a residue was obtained, which was taken up in H_2_O and successively partitioned with petroleum ether (PE, three times) and ethyl acetate (EtOAc, three times). The EtOAc layer (1200 g) was absorbed on macroporous resin (D101) and subjected to column chromatography [MeOH:H_2_O (1:4, 3:2, 4:1, 5:0)] to give four main fractions (Fr.1–Fr.4). Fr.1 (400 g) was subjected to silica gel column chromatography with CH_2_Cl_2_/MeOH (100:1, 50:1, 20:1, 5:1) and divided into four fractions. The 50:1 fraction was applied to a chromatography column for isolation of **3** (5 mg). The 20:1 fraction was treated by Sephadex LH-20 (MeOH) and divided into three subfractions (Fr.A, Fr.B, and Fr.C). Fr.A was applied to a chromatography column for isolation of **2** (8.8 mg). Fr.B was subjected to semipreparative HPLC (MeOH/H_2_O, 40% to yield compound **6** (31 mg, t_R_ = 9.85 min) and compound **7** (26 mg, t_R_ = 10.65 min)). The 5:1 fraction was also subjected to silica gel column chromatography and three subfractions were obtained (PE/acetone: 5:1, 2:1, 1:2). Compound **4** (6.5 mg) and **5** (8.5 mg) were isolated from the 2:1 fraction by repeated silica gel column chromatography (CH_2_Cl_2_/MeOH). Reverse phase silica gel (MeOH/H_2_O, step gradient) was used to treat the 1:2 fraction to give **1** (20.1 mg).

Methyl ganoderate G1 (**1**): [α]^25^_D_ −90 (c 0.11, MeOH); UV (MeOH); λmax (log ε): 253 (4.05) nm. IR (KBr) vmax 3425, 2972, 1720, 1629, 1461 cm^-1^; ^1^H and ^13^C-DEPT data: see Table 1. HRESIMS *m/z* 571.3241 (calcd for C_31_H_48_O_8_Na 571.3247).

### 4.4. Bioactivity Evaluation

#### 4.4.1. ABTS Radical Cation Scavenging Activity

ABTS radical cation scavenging activity was assayed according to instructions by the Beyotime Institute of Biotechnology. The stock solutions included ABTS solution and oxidant solution. The working solution was prepared by mixing the two stock solutions in equal quantities and allowing them to react for 16 h at room temperature in the dark. The solution was then diluted by mixing 1 mL working solution with 90 ml 80% ethanol in order to obtain an absorbance of 0.7 ± 0.05 at 734 nm. A fresh ABTS solution was prepared for each assay. Samples (10 μL) with a concentration range of 0.05–3.00 mM were mixed with 200 μL of fresh ABTS solution and the mixture was left at room temperature for 6 min. The absorbance was then measured at 734 nm. Trolox was used as a reference compound. The radical scavenging activity of each sample was expressed in terms of the EC_50_ (the effective concentration at which ABTS^.+^ radicals were scavenged by 50%), which was calculated from the log–dose inhibition curve.

#### 4.4.2. Oxygen Radical Absorbance Capacity Assay (ORAC Assay)

The ORAC assay was carried out based on the previously described procedure with slight modification [36]. In brief, the sample/blank (175 μL) was dissolved in phosphate buffer saline (PBS) at the concentration of 160 μg/mL at pH 7.4. The trolox standard was prepared in serial dilutions starting from 75 mM. Standard 96-well black microplates were used for the assay, and 25 μL each of the samples, standard (trolox), blank (solvent/PBS), or positive control (quercetin) were added to the wells. Fluorescent sodium salt solution was added at 150 μL per well, followed by incubation at 37 °C for 45 min. The total volume of each well was made up to 200 μL by adding 2, 20-azobis-(2-amidinopropane) dihydrochloride (AAPH) solution. Fluorescence value was recorded at 37 °C (excitation at 485 nm, emission at 535 nm) using a fluorescence spectrophotometer (Infinite M200 PRO) equipped with an automatic thermostatic autocell holder. Data were collected every 2 min for 2 h and the data analysis was subsequently done by calculating the differences of AUC between the blank and the sample. Results were expressed as μmol of Trolox Equivalent/μmol of pure compound. 

#### 4.4.3. Cell Culture

The human neuroblastoma cell line (SH-SY5Y) was obtained from Shanghai Institute of Cell Biology, Chinese Academy of Sciences. SH-SY5Y cells were cultured in MEM/F12 (1:1) medium supplemented with 10% FBS and 1% penicillin–streptomycin at 37 °C and 5% CO2. 

#### 4.4.4. Cytoprotective Effects of Compounds **1**–**7** in SH-SY5Y

Compounds **1**–**7** were dissolved in DMSO for use (DMSO in each well was less than 0.1%). Before treatment, 100 μL SH-SY5Y cells were seeded on a 96-well plate at the density of 1 × 10^5^ cells/mL and cultured for 24 h. The medium was changed to 99 μL fresh medium containing 200 μM H_2_O_2_, and then cells were treated with 1 μL different concentrations of compounds **1**–**7** for 12 h. CCK-8 solution (10 μL) was added to every well, and coincubated for 2 h at 37 °C. The absorbance was measured at 450 nm using a TECAN microplate reader.

#### 4.4.5. Neuroprotective Effects of Compounds **1**, **6**, and **7** in SH-SY5Y

Compounds **1**, **6**, and **7** were dissolved in DMSO for use (DMSO in each well was less than 0.1%). Before treatment, 100 μL SH-SY5Y cells were seeded on a 96-well plate at the density of 1 × 10^5^ cells/mL and cultured for 24 h. The medium was changed to 99 μL fresh medium containing 25 μM aged Aβ_25__–35_, and then cells were treated with 1 μL different concentrations of compounds **1**, **6**, and **7** for 24 h. CCK-8 solution (10 μL) was added to every well, and coincubated for 2 h at 37 °C. The absorbance was measured at 450 nm using a Bio-Rad microplate reader.

#### 4.4.6. Measurement of Intracellular ROS Level

The generation of intracellular reactive oxygen species (ROS) was measured using the DCFH-DA method [37]. Briefly, SH-SY5Y cells were cultured in 96-well plates (1 × 10^4^ cells per well). Drug treatment, H_2_O_2_ stimulation, and Aβ_25__–__35_ stimulation were carried out as described in Section 4.4.4 and Section 4.4.5. The cells were washed with warm PBS and were incubated with DCFH-DA for 30 min at 37 °C in darkness. After the cells were washed twice with PBS, the fluorescence intensity was measured at an excitation wavelength of 485 nm and an emission wavelength of 538 nm. The level of intracellular ROS was expressed as a percentage of value against the nontreated control group.

#### 4.4.7. Statistical Analysis

Data analysis was performed using GraphPad Prism 6.0 software (GraphPad Software, San Diego, USA). All results are presented as the mean ± S.D. and a two-tailed test or a one-way analysis of variance (ANOVA) was used to determine the statistical significance. Differences were considered to be significant for *p*-value <0.05.

## Figures and Tables

**Table 1:** NMR spectroscopic data for compound **1** in CDCl_3_.

Position	1		Position	1	
	*δ_H_* (J in Hz)	*δ_C_*		*δ_H_* (J in Hz)	*δ_C_*
1	1.68 m	34.61	16	1.79 m	36.25
	2.75 m			2.50 m	
2	2.15 m	28.28	17	1.81 m	48.10
			18	0.95 s	17.12
3	3.23 dd (10.8, 5.5)	78.19	19	1.26 s	19.38
4	-	38.61	20	2.15 m	32.63
5	2.95 m	49.07	21	0.89 d (6.4)	19.62
6	1.64 m	27.80	22	1.8 m	49.72
	2.13 m				
7	4.57 dd (10.3, 7.2)	69.52	23	-	208.53
8	-	157.78	24	2.47 m	46.72
9	-	141.96		2.83 m	
10	-	38.52	25	2.08 m	34.61
11	-	199.73	26	-	176.26
12	4.14 d (2.3)	77.22	27	1.19 d (7.1)	17.09
13	-	47.12	28	1.04 s	28.15
14	-	53.90	29	0.86 s	15.67
15	4.76 t (7.8)	72.48	30	1.27 s	19.48
			COOMe	3.69 s	51.91

**Table 2:** ABTS^.+^ radical scavenging activity and ORAC values of compounds **1**–**7** (*n* = 6).

Samples	Antioxidant Capacity	
	ABTS Radical Scavenging Assay(EC_50_ ± SD, mM)	ORAC Value (μmol TE/ μmol)(mean ± SD)
**1**	1.12 ± 0.17	1.35 ± 0.25
**2**	2.32 ± 0.16	0.36 ± 0.14
**3**	2.18 ± 0.15	0.25 ± 0.18
**4**	1.64 ± 0.18	0.51 ± 0.12
**5**	1.85 ± 0.14	0.59 ± 0.18
**6**	0.59 ± 0.15	5.42 ± 0.20
**7**	0.27 ± 0.05	7.24 ± 0.15
Trolox	0.42 ± 0.03	-
Quercetin	-	7.78 ± 0.27

## Supplementary Materials

The following are available online at https://www.mdpi.com/1420-3049/24/23/4353/s1, Figure S1. ^1^H NMR spectrum of compound **1** in CDCl_3_; Figure S2. ^13^C NMR spectrum of compound **1** in CDCl_3_; Figure S3. DEPT spectrum of compound **1** in CDCl_3_; Figure S4. ^1^H-^1^H COSY spectrum of compound **1** in CDCl_3_; Figure S5. HSQC spectrum of compound **1** in CDCl_3_; Figure S6. ROESY spectrum of compound **1** in CDCl_3_; Figure S7. HMBC spectrum of compound **1** in CDCl_3_.
