# Supplementary material for: Triterpenes and Aromatic Meroterpenoids with Antioxidant Activity and Neuroprotective Effects from Ganoderma lucidum
Source: Molecules. 2019 Nov 28;24(23):4353. doi: 10.3390/molecules24234353 (PMC6930543; doi:10.3390/molecules24234353)
Supplement: Supplementary file 1 [file molecules-24-04353-s001.pdf]

Supporting Information

Triterpenes and aromatic meroterpenoids with antioxidant activity and neuroprotective effects from *Ganoderma lucidum*

Cuifang Wang[1,*], Xuemin Liu[2], Chenlei Lian [2], Jiaying Ke [1] and Jieqing Liu [2,*]

[1] College of Oceanology and Food Science, Quanzhou Normal University, Quanzhou 362000, P.R. China; kejiaying2003@e-mail.com (J.-Y.K.)
[2] School of Medicine, Huaqiao University, Quanzhou 362021, P.R. China; 1329948465@qq.com (X.-M. L.); liancl@hqu.edu.cn (C.-L.L.);
* Correspondence: wangcuifang@qztc.edu.cn (C.-F.W.); liujieqing@hqu.edu.cn (J.-Q.L.); Tel.: +86-0595-2297-9207 (C.-F.W.)

Figure S1. ^{1}H NMR spectrum of compound **1** in $CDCl_3$ 2

Figure S2. ^{13}C NMR spectrum of compound **1** in $CDCl_3$ 3

Figure S3. DEPT spectrum of compound **1** in $CDCl_3$ 4

Figure S4. ^{1}H-^{1}H COSY spectrum of compound **1** in $CDCl_3$ 5

Figure S5. HSQC spectrum of compound **1** in $CDCl_3$ 6

Figure S6. ROESY spectrum of compound **1** in $CDCl_3$ 7

Figure S7. HMBC spectrum of compound **1** in $CDCl_3$ 8

Compound characterization data of **2-7** 9

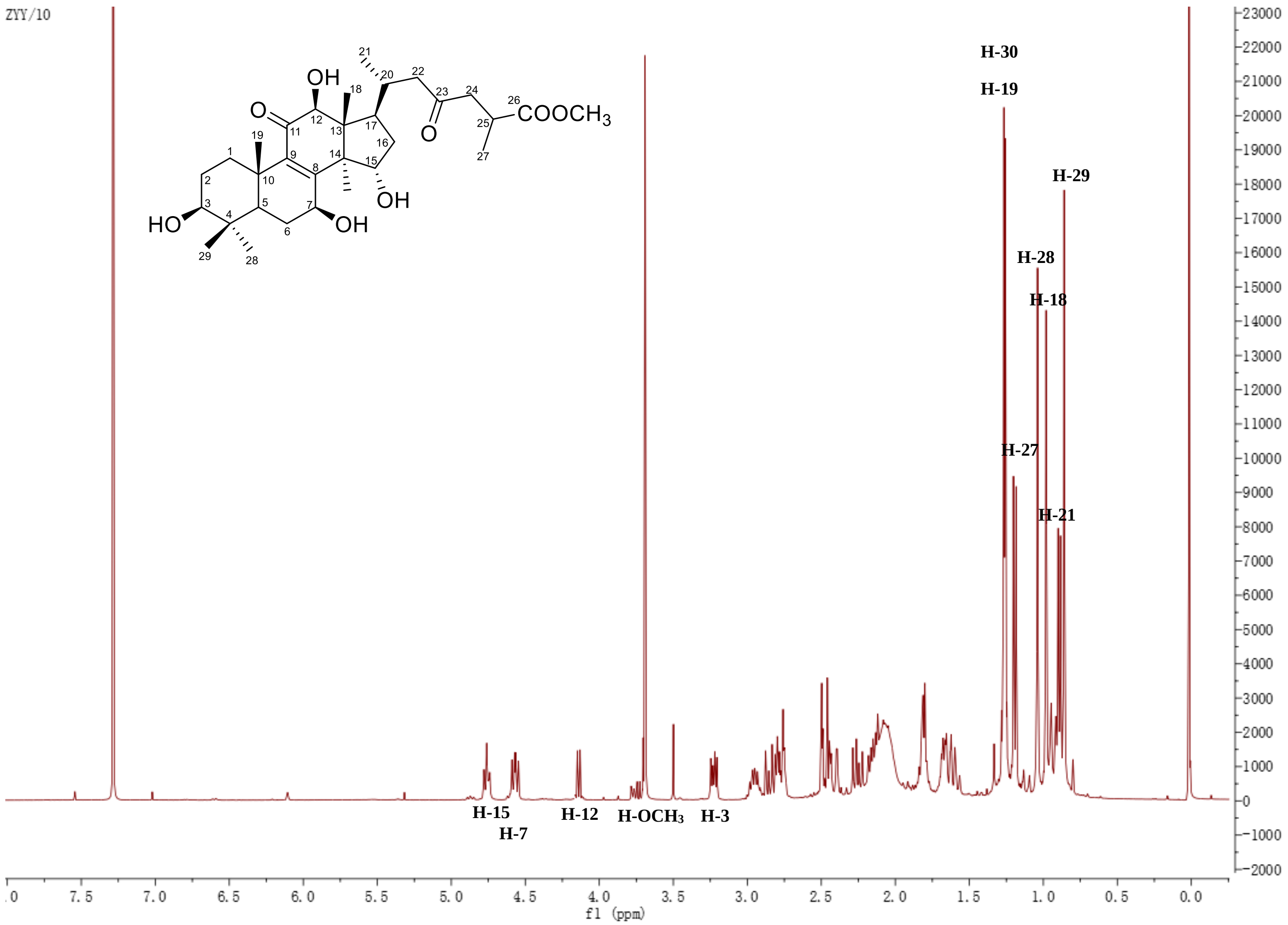

Figure S1. ^{1}H NMR spectrum of compound **1** in $CDCl_3$

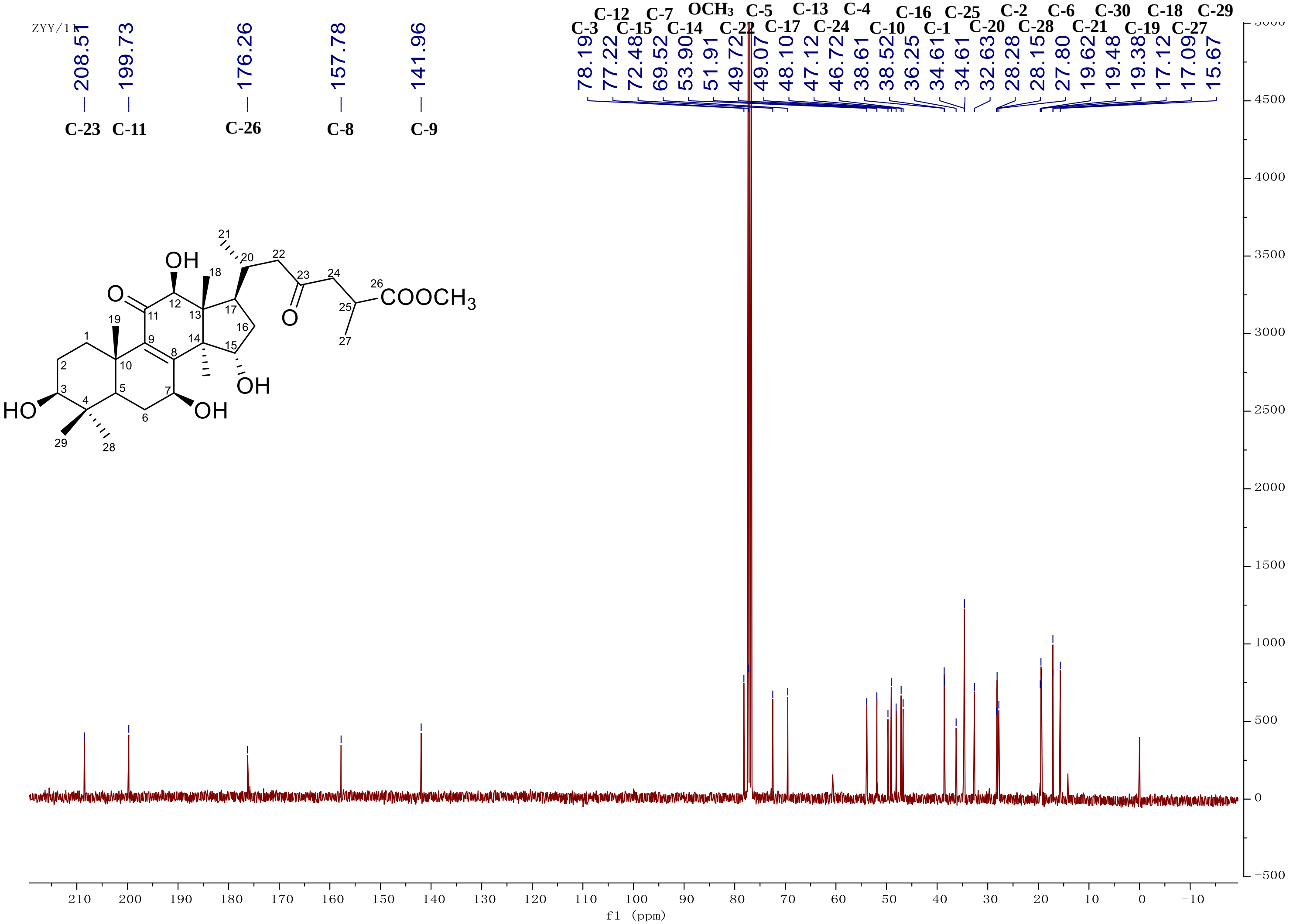

Figure S2. ^{13}C NMR spectrum of compound **1** in $CDCl_3$

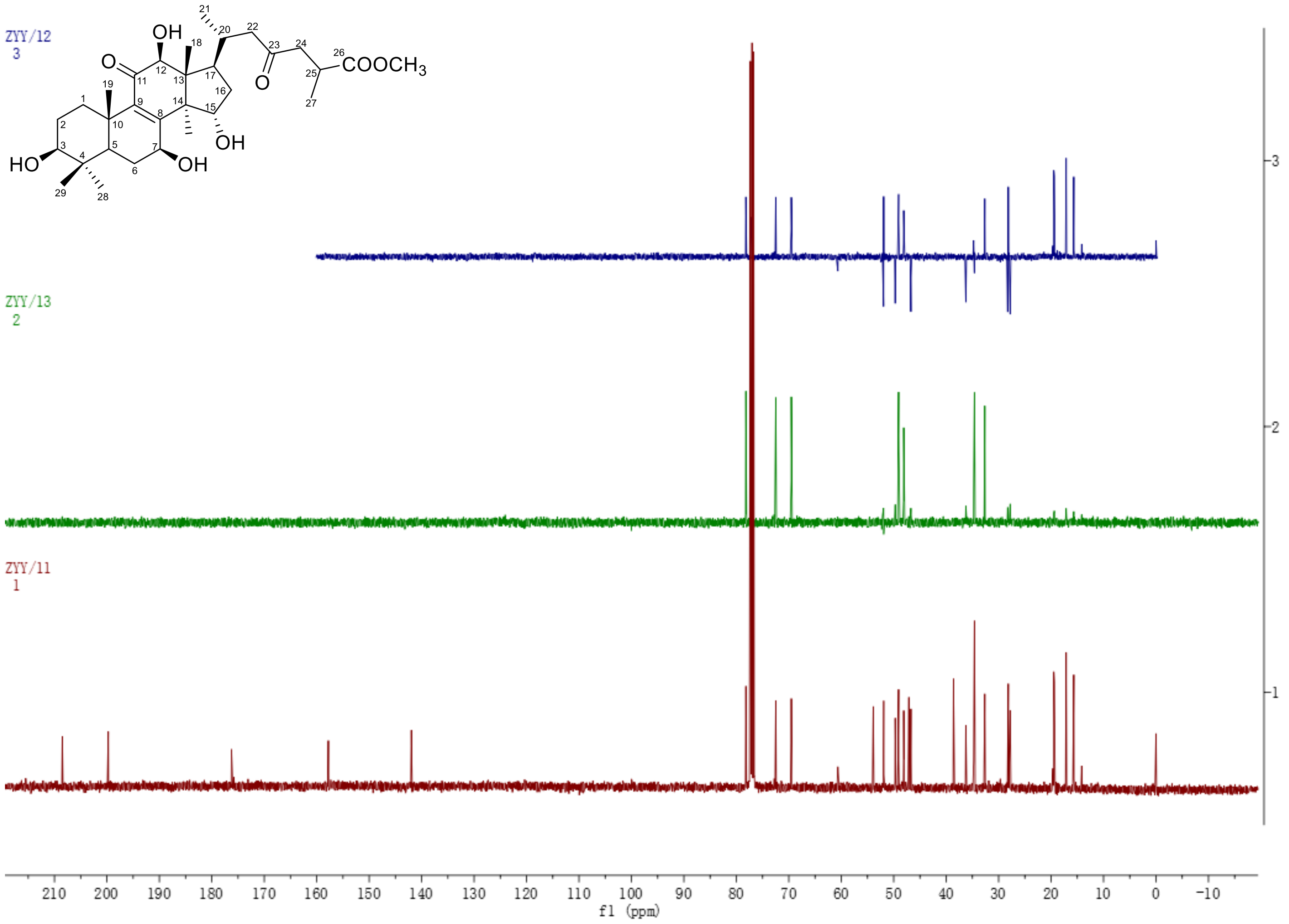

Figure S3. DEPT spectrum of compound **1** in $CDCl_3$

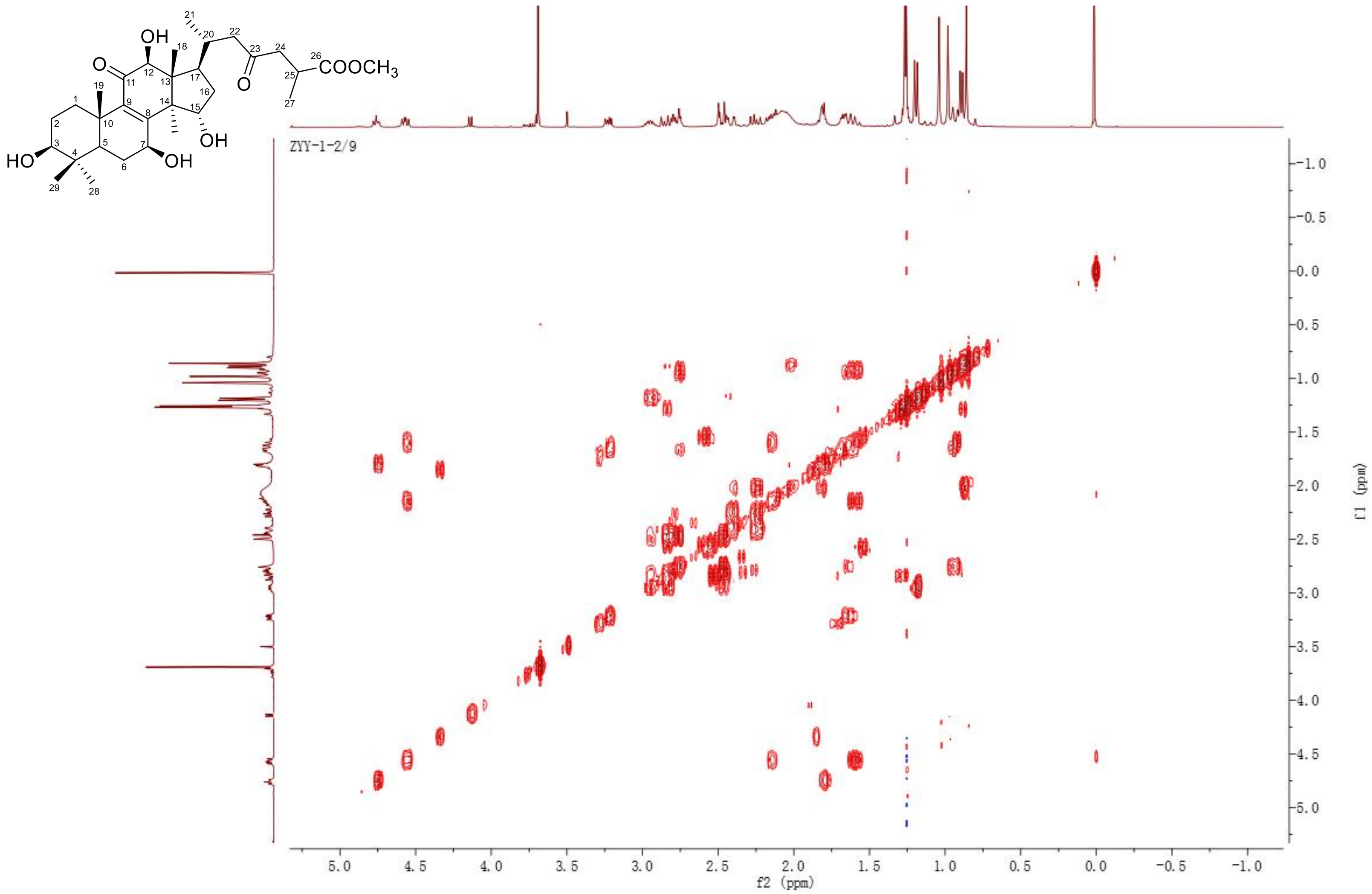

Figure S4. ^{1}H-^{1}H COSY spectrum of compound **1** in $CDCl_3$

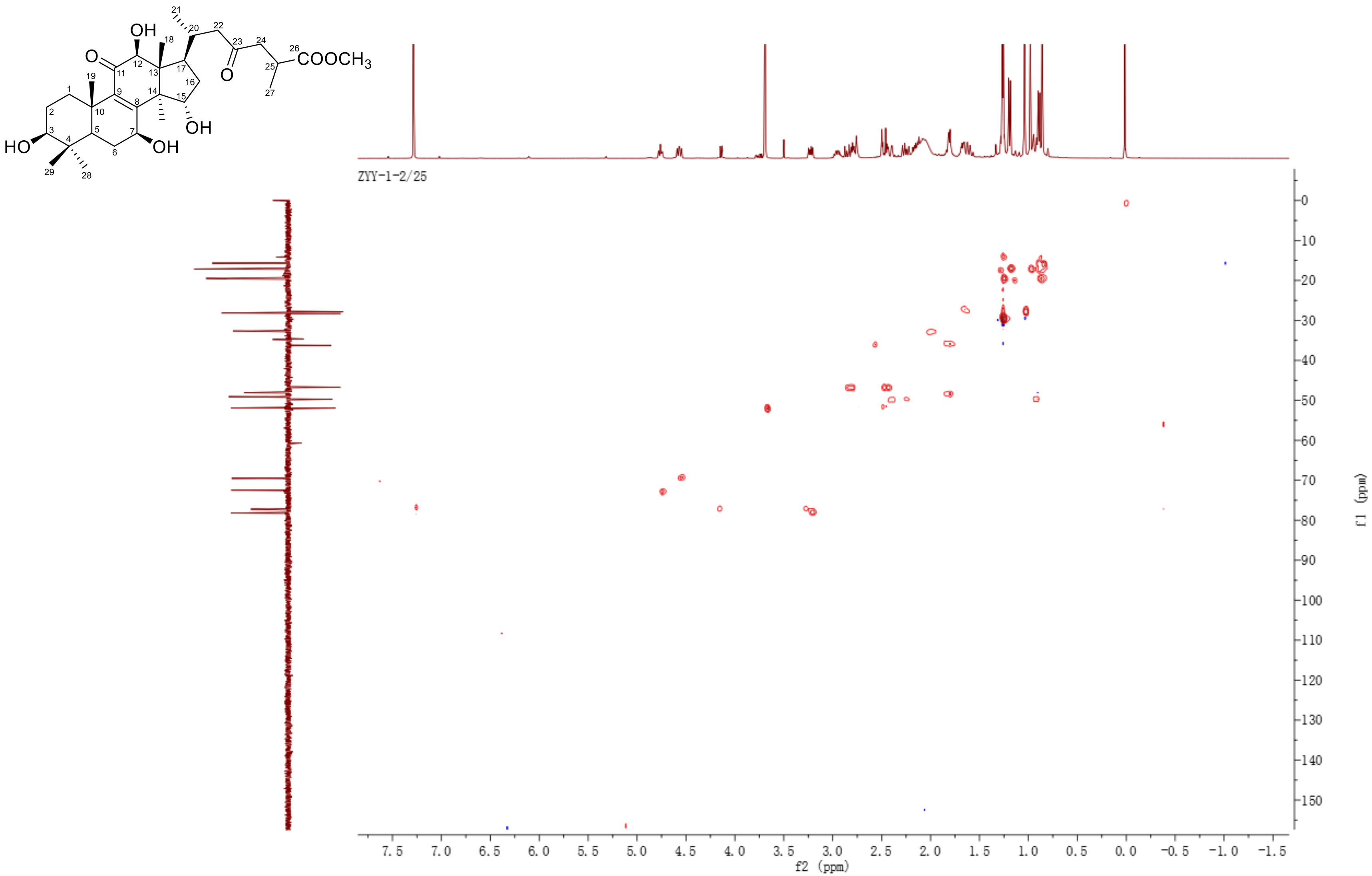

Figure S5. HSQC spectrum of compound **1** in $CDCl_3$

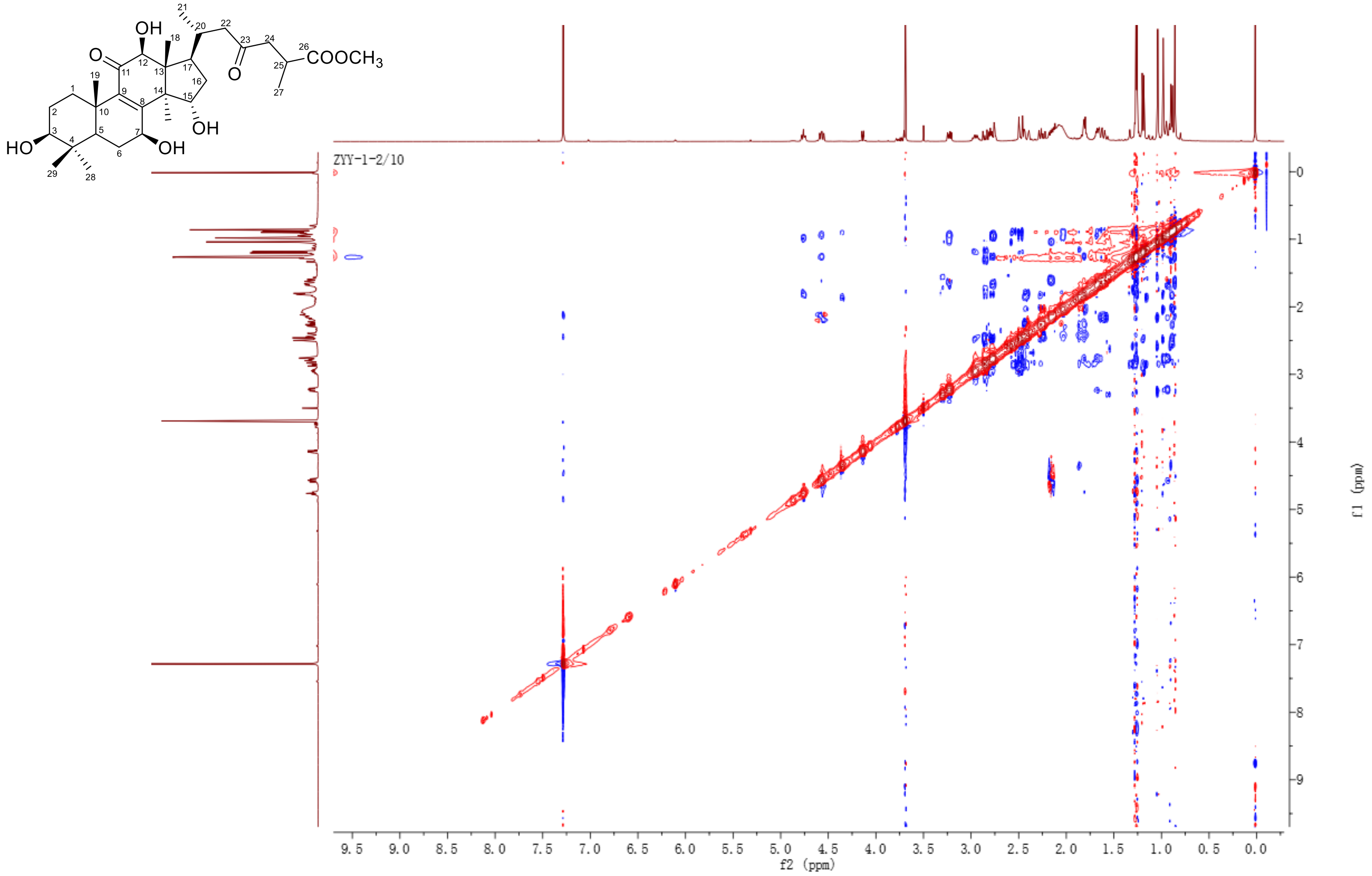

Figure S6. ROESY spectrum of compound **1** in $CDCl_3$

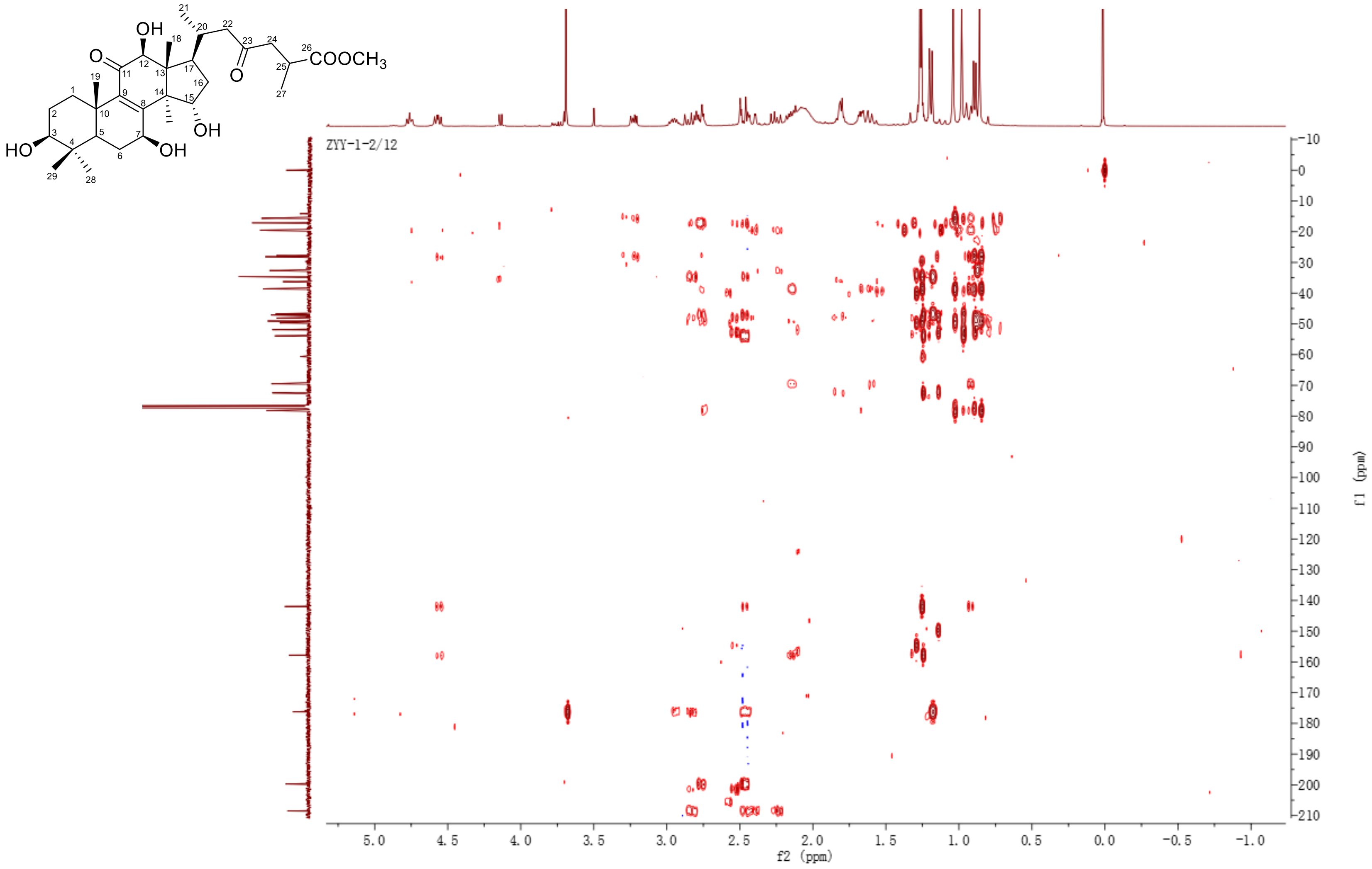

Figure S7. HMBC spectrum of compound **1** in $CDCl_3$

Compound characterization data

Ganoderic acid D2 (**2**): white powder, ESI-MS *m/z*: 529 $[M+H]^+$, $C_{30}H_{42}O_8$. ^1H NMR (500 MHz, DMSO) δ_H 4.84–4.74 (m, H-7), 4.32 (s, H-12), 1.32 (s, H-30), 1.23 (s, H-19), 1.04 (s, H-28/29), 1.01 (s, H-27), 1.00 (s, H-18), 0.76 (s, H-21); ^{13}C NMR (125 MHz, DMSO) δ_C: 215.99 (C-15), 209.79 (C-3), 200.83 (C-23), 159.26 (C-11), 140.25 (C-26), 78.51 (C-12), 64.93 (C-7), 59.18 (C-14), 51.07 (C-4), 48.50 (C-22/24), 48.46 (C-8), 46.66 (C-9), 46.57 (C-13), 46.34 (C-5), 38.67 (C-16), 37.67 (C-10),35.74 (C-17), 35.11 (C-1), 34.27 (C-2), 32.13 (C-20), 28.52 (C-6), 28.14 (C-25), 26.70 (C-30), 24.19 (C-29), 21.99 (C-28), 21.09 (C-18), 18.39 (C-21), 17.85 (C-27), 12.84 (C-19).

Ganoderic acid H (**3**): white powder, ESI-MS m/z: 571 $[M+H]^+$, $C_{32}H_{44}O_9$. ^1H NMR (500 MHz, DMSO) δ_H: 5.65 (s, H-12); 3.29 (dd, *J* = 11.4, 4.9 Hz, H-3), 2.26 (s, -COC$\underline{H}_3$), 1.75 (s, H-30), 1.35 (s, H-19), 1.24 (d, J = 7.2 Hz, H-27), 1.05 (s, H-18), 1.00 (d, J = 6.0 Hz, H-21), 0.90 (s, H-29), 0.84 (s, H-28); ^{13}C NMR (125 MHz, DMSO) $\delta_{C:}$ 208.96 (C-23), 206.55 (C-15), 199.17 (C-11), 194.73 (C-7), 177.13 (C-26),169.81 (-$\underline{C}OCH_3$), 151.34 (C-8), 145.96(C-9), 79.28(C-12), 75.88 (C-3), 58.46 (C-30), 51.11 (C-5), 48.33 (C-14), 48.00 (C-22), 47.97 (C-13), 46.21 (C-24), 44.50 (C-4), 39.17 (C-10), 37.78 (C-16), 36.86 (C-6), 34.70 (C-17), 33.11 (C-1), 29.28 (C-20), 28.17 (C-29), 27.53 (C-2), 21.70 (C-28), 21.11 (C-30), 21.00 (-CO$\underline{C}H_3$), 18.01 (C-19), 17.33 (C-21), 16.27 (C-27), 12.22 (C-18).

Lucidumol B (**4**): white powder, ESI-MS *m/z*: 459 $[M+H]^+$, $C_{30}H_{50}O_3$, ^1H NMR (500 MHz, DMSO) δ_H: 5.47 (d, J = 6.2 Hz, H-7), 5.33 (d, *J* = 6.1 Hz, H-11), 4.42 (dd, J = 14.3, 5.0 Hz, H-24), 4.28 (d, J = 5.7 Hz, H-3), 1.04 (s, H-30), 0.98 (s, H-19), 0.91 (d, *J* = 5.6 Hz, H-26/27), 0.87 (d, *J* = 6.5 Hz, H-21), 0.84 (s, H-29), 0.78 (s, H-28), 0.53 (s, H-18); ^{13}C NMR (125 MHz, DMSO) δc: 146.14 (C-9), 142.64 (C-8), 120.62 (C-7), 116.23 (C-11), 78.87 (C-24), 77.27 (C-3), 72.20 (C-25), 50.96 (C-17), 50.34 (C-14), 49.37 (C-5), 43.75 (C-13), 38.80 (C-4), 37.73 (C-12), 37.42 (C-10), 36.63 (C-20), 35.85 (C-1), 33.85 (C-22), 31.56 (C-15), 28.78 (C-29), 28.15 (C-23), 27.98 (C-16), 27.85 (C-2), 26.80 (C-28), 25.96 (C-27), 24.84 (C-26), 23.10 (C-6), 23.02 (C-30), 19.05 (C-19), 16.58 (C-21), 15.95 (C-18).

Ganoderiol B (**5**): white powder, ESI-MS *m/z:* 441 $[M+H]^+$, $C_{30}H_{50}O_2$. ^1H NMR (500 MHz, $CDCl_3$) δ_H: 5.49 (d, *J* = 6.3 Hz, H-24), 5.42 (td, *J* = 7.2, 1.4 Hz, H-7), 5.33 (d, *J* = 6.3 Hz, H-11), 4.02 (d, *J* = 1.2 Hz, H-26), 3.27 (dd, *J* = 11.4, 4.4 Hz, H-3), 1.69 (s, H-27), 1.03 (s, H-30), 1.00 (s, H-19), 0.94 (d, *J* = 6.5 Hz, H-21), 0.90 (d, *J* = 1.4 Hz, H-26/27), 0.59 (s, H-18); ^{13}C NMR (125 MHz, $CDCl_3$) δc: 145.91(C-9), 142.65(C-8), 134.34(C-25), 127.01(C-24), 120.22(C-7), 116.27(C-11),

78.97(C-3), 69.09(C-26), 50.92(C-17), 50.32(C-14), 49.12(C-5), 43.78(C-13), 38.71(C-4), 37.83(C-10), 37.37(C-12), 36.09(C-20), 35.93(C-22), 35.72(C-1), 31.51(C-15), 28.15(C-16), 27.92(C-2), 27.80(C-23), 25.58(C-28), 24.54(C-29), 23.01(C-6), 22.76(C-30), 18.41(C-19), 15.80(C-21), 15.67(C-18), 13.65(C-27).

Lingzhine E (**6**): yellowish solid, ESI-MS *m/z*: 307 $[M+H]^+$, $C_{16}H_{18}O_6$. ^{1}H NMR (500 MHz, DMSO) δ_H: 6.86 (d, *J* = 3.1 Hz, H-3), 6.83 (dd, *J* = 8.6, 3.2 Hz, H-5), 6.66 (d, *J* = 8.6 Hz, H-6), 3.50 (dd, *J* = 18.0, 9.9 Hz, H-2'a), 1.55 (s, H-9'); ^{13}C NMR (125 MHz, DMSO) δc: 152.41 (C-1), 125.32 (C-2), 115.51 (C-3), 149.67 (C-4), 125.82 (C-5), 119.34 (C-6), 205.66 (C-1'), 37.91 (C-2'), 50.71 (C-3'), 75.73 (C-4'), 29.43 (C-5'), 119.23 (C-6'), 132.92 (C-7'), 69.15 (C-8'), 18.78 (C-9'), 176.58 (C-10').

Lingzhine F (**7**): yellowish solid, ESI-MS *m/z*: 307 $[M+H]^+$, $C_{16}H_{18}O_6$. ^{1}H NMR (500 MHz, DMSO) δ_H: 6.96 (d, *J* = 3.0 Hz, H-3), 6.86 (dd, *J* = 8.8, 3.0 Hz, H-5), 6.68 (d, *J* = 8.7 Hz, H-6), 3.80 (dd, *J* = 18.0, 10.9 Hz, H-2'a), 1.60 (s, H-9'); ^{13}C NMR (125 MHz, DMSO) δc: 152.05 (C-1), 126.61 (C-2), 115.55 (C-3), 149.52 (C-4), 125.92 (C-5), 119.75 (C-6), 205.02 (C-1'), 36.66 (C-2'), 50.09 (C-3'), 74.59 (C-4'), 26.01 (C-5'), 119.75 (C-6'), 132.89 (C-7'), 69.34 (C-8'), 18.77 (C-9'), 175.29 (C-10').
